# The Impact of Brexit on the Pharmaceutical Supply Chain of the United Kingdom: Scoping Review Protocol

**DOI:** 10.2196/17684

**Published:** 2020-09-23

**Authors:** Madison Milne-Ives, Ching Lam, Michelle van Velthoven, Edward Meinert

**Affiliations:** 1 Digitally Enabled PrevenTative Health Research Group Department of Paediatrics University of Oxford Oxford United Kingdom; 2 Department of Primary Care and Public Health Imperial College London London United Kingdom; 3 Centre for Health Technology Faculty of Health University of Plymouth Plymouth United Kingdom

**Keywords:** Brexit, drug industry, pharmaceutical supply

## Abstract

**Background:**

The continuing uncertainty around Brexit has caused concern in the pharmaceutical industry and among health care professionals and patients. The exact consequences of Brexit on the pharmaceutical supply chain in the United Kingdom will depend on whether a deal is reached and what it entails, but it is likely to be affected by the withdrawal of the United Kingdom from the European Union. Regulatory issues and delays in supply have the potential to negatively affect the ability of UK residents to receive an adequate and timely supply of necessary medicines.

**Objective:**

The purpose of this protocol is to provide an overview and critical analysis of current perspectives on the effect of Brexit on the UK pharmaceutical supply chain.

**Methods:**

The PRISMA-ScR (Preferred Reporting Items for Systematic reviews and Meta-Analyses extension for Scoping Reviews) guidelines will be used to structure this protocol. A systematic search of MEDLINE, EMBASE, PsycINFO, Healthcare Management Information Consortium (HMIC), Cochrane, Web of Science, Business Source Complete, EconLit, and Economist Intelligence Unit will be conducted, as well as a Google and Nexis.UK search for grey literature such as reports, opinion pieces, and press releases. Two reviewers will independently screen the titles and abstracts of identified references and select studies according to the eligibility criteria. Any discrepancies will then be discussed and resolved. One reviewer will extract data from the included studies into a standardized form, which will be validated by a second reviewer. Risk of bias will be assessed using the Cochrane Collaboration Risk of Bias tool for any randomized controlled trials; quality will be assessed using the relevant Critical Appraisal Skills Programme (CASP) checklists; and grey literature will be assessed using the Authority, Accuracy, Coverage, Objectivity, Date, Significance (AACODS) checklist. Outcomes include the agreement between sources on the potential, likelihood, and severity of the consequences of Brexit on the UK pharmaceutical supply chain.

**Results:**

Results will be included in the scoping review, which will be published in 2020.

**Conclusions:**

This scoping review will summarize the currently expected consequences of Brexit on the UK pharmaceutical supply chain.

**International Registered Report Identifier (IRRID):**

PRR1-10.2196/17684

## Introduction

The long uncertainty around whether and how the United Kingdom will leave the European Union (“Brexit”) makes it difficult to predict the exact consequences for the health care system at the time of writing (December 31, 2019). These consequences will depend on what trade deals are arranged between the United Kingdom and European Union (EU), how involved the United Kingdom will remain in EU health care systems, the transition time allowed for companies to adapt to new regulations, and which EU regulations will be adopted into British law [1]. No matter the exact conditions of Brexit, however, changes to the relationship between the United Kingdom and EU are likely to have implications for the authorization and accessibility of medicines [2]. The uncertainty around how Brexit will occur has already caused concern in the pharmaceutical industry and preparation for a worst-case scenario [3]. In 2017, pharmacists were already reporting that this restructuring of the supply chain was causing medicine shortages [4].

The pharmaceutical supply chain is likely to be affected by Brexit at numerous stages. The United Kingdom is currently a member of the European Medicines Agency (EMA), which facilitates the single market for medicines in the EU [5]. If the United Kingdom leaves with a deal, there will be an important transition period that should prevent large disruptions of medicine supplies [4]. If there is no deal, the United Kingdom will immediately not be subject to EU law or EMA regulations, which could affect supply [5]. The extent of United Kingdom involvement in EU pharmaceutical activities will affect drug production, authorization, regulation, trade, health and safety monitoring, and research [3,4,6].

Depending on the post-Brexit UK-EU relationship, pharmaceutical companies might need separate centers in the United Kingdom and EU to test and release medicines, which could incentivize moving their headquarters from the United Kingdom to the EU, as the EMA already did [3,6]. The EMA currently approves marketing authorizations for the entire EU, but if the UK Medicines and Health products Regulatory Agency (MHRA) is unwilling to accept EMA decisions, drugs will need to undergo an additional authorization process to reach UK markets [7]. This will likely mean increased costs and delays in medicines becoming available in the United Kingdom and could deter companies from selling their medicines in the UK market altogether [3,4,6]. Additionally, if the United Kingdom is unwilling to accept regulatory requirements from other agencies, such as the EMA or the Food and Drugs Administration (FDA) of the United States, a new regulatory system will need to be developed [6].

If the United Kingdom leaves the EU without a deal, World Trade Organization (WTO) rules will come into effect [8], and tariffs on the trade of medicines will also increase the costs of drug production. Even with a trade deal, changes in the supply chain and potential costs of customs would increase costs [3]. Already during the transition period, no UK representatives can participate in EMA meetings [9]. A disconnect between the MHRA and the EMA could also limit data sharing. This would affect the United Kingdom’s ability to effectively monitor the safety and efficacy of postapproval medicines [3,4]. The EU is also a leader in the monitoring of counterfeit drugs and global supply chains [10]. After Brexit, the United Kingdom could still be included but would have no influence to direct this monitoring [6]. This loss of influence and prestige in the pharmaceutical industry has already begun with the move of the EMA headquarters from London to Amsterdam [6].

Research is another area of concern for UK pharmaceutical companies and scientists, as not being an EU member state means the United Kingdom may no longer be eligible for EU research funding programs like Horizon 2020 [3]. Lack of inclusion in the EU could also affect clinical research trials. Pharmaceutical companies would have to register trials separately in the United Kingdom and the EU, and would require another authorization to recruit UK patients. These extra hurdles might make companies unwilling to use UK patients, which would deny patients early access to new treatments and make results less generalizable to UK populations [3,11].

The different forms that Brexit could take will influence its effect on the pharmaceutical supply chain. If a deal is established that allows the United Kingdom access to the single market, similar to the European Economic Area (EEA), or that keeps the MHRA integrated with EU health activities and negotiates free trade agreements with EU countries, the costs and availability of medicines are unlikely to be seriously affected, and the most severe consequence would likely be the loss of the United Kingdom’s prestige in the pharmaceutical industry [1,6]. If the United Kingdom sets up free trade agreements but the MHRA is not involved with the EMA, there could be delays in market authorization approvals, loss of expertise in postapproval safety monitoring; delays in detecting and managing risks because of reduced communication and data sharing; and increased production costs to cover duplicate batch testing in the United Kingdom and EU [1,6]. A Brexit that applies WTO rules to UK-EU trade could result in medicine shortages because of the significant changes to the supply chain and largely increased costs due to tariffs [1,6]. Essentially, the more disconnected the United Kingdom becomes from the EU, the more severe the changes to the pharmaceutical supply chain and the potential health consequences [6]. The uncertainty of Brexit has meant that many companies are already preparing for worst-case scenarios, which means that consequences could be felt before Brexit occurs [1].

The uncertainty around Brexit has led to a variety of opinions, possible scenarios, and dire warnings of medicine shortages. However, a search of PROSPERO (International Prospective Register of Systematic Reviews) for “Brexit” and “Pharmaceutical” returns no results, and neither does a search for “Brexit” alone. This lack of reviews means that there is a need to aggregate all of the opinions and perspectives on the effect that Brexit will have on the pharmaceutical industry and assess the expected consequences and levels of concern about them. The scope of this review is adopted from a paper that uses the WHO Health System Framework [12] to structure its evaluation of how Brexit could affect UK health services [6]. Therefore, this review will focus on two main research questions. First, what are the potential consequences of Brexit for the UK pharmaceutical supply chain (assessed according to the WHO system building blocks)? Second, to what extent is there agreement on the likelihood and severity of these potential consequences?

## Methods

### Overview

The PRISMA-ScR (Preferred Reporting Items for Systematic Reviews and Meta-Analyses Protocols Extension for Scoping Reviews) guidelines will be used to structure the review [13]. The scoping review will conduct a literature search, article selection, data extraction, quality appraisal, data analysis, and data synthesis.

### Eligibility

#### Inclusion Criteria

We will include academic and grey literature published in 2016 or later. The year 2016 was chosen because that was the year of the Brexit referendum [14] and because the process of Brexit has been so uncertain that more recent publications are more likely to reflect current expected consequences. Both academic and grey literature are being considered because nonacademic sources such as company and institute reports and news articles are likely to have relevant information. Publications that discuss at least one potential effect of Brexit on the UK pharmaceutical supply chain will be included.

#### Exclusion Criteria

We will exclude publications that are not written in English and publications that do not focus on the effect of Brexit on the UK pharmaceutical supply chain specifically. We will focus exclusively on the United Kingdom to narrow the scope of the study. Publications, particularly from the grey literature, will also be excluded if they are not of high quality. The quality of grey literature will be assessed using the AACODS checklist [15], which examines whether certain criteria are present in the source. A value of 1 will be assigned to a “yes,” and if the number of “yes” responses is less than half of the total possible, the source will be excluded [16].

### Search Strategy

The following databases will be searched: MEDLINE, EMBASE, PsycINFO, Healthcare Management Information Consortium (HMIC), Cochrane, Web of Science, Business Source Complete, EconLit, Economist Intelligence Unit, and Nexis.UK. These will be accessed through the University of Oxford Search Oxford Libraries Online (SOLO) interface when possible. In addition, a Google search for grey literature such as opinion pieces, institute reports, press releases, and blog posts will be conducted. A combination of effort-bounded and evidence exhaustion criteria will be used to limit the Google search: the first 100 results will be screened, and if those near the end of the list are still providing new, relevant information, screening will continue up to the 200th result, or until sources cease to provide new information [16]. Key search terms were identified in a preliminary review of the literature, search strings were constructed, and databases were chosen in consultation with a librarian. Table 1 shows the search concept and keywords to be searched for this review. Databases will be searched for items published from the beginning of 2016 to the date of search.

**Table 1:** Search terms.

Category	MeSH terms	Keywords (in title or abstract)
Brexit	European Union, United Kingdom	Brexit OR United Kingdom OR Britain OR ((“EU” OR “European Union”) AND “single market”) OR “Article 50” OR ((leave OR withdraw* OR exit OR remain OR stay) ADJ4 (“European Union” OR “EU” OR “EEA” OR “European Economic Area”)) OR “Post-Brexit”
Pharmaceuticals	Drug industry, pharmaceutical preparations, pharmaceutical societies, pharmaceutical economics, drug approval, United States Food and Drug Administration	((Pharma* OR drug* or medic*) ADJ4 (industr* OR compan* OR supply OR sector OR production OR approval OR deliver* OR regulat* OR preparation* OR societ* OR econom* OR manufactur* OR shortage* OR stockpil* OR stock-pil*)) OR “EMA” OR “European Medicines Agency” OR “MHRA” OR “Medicines and Health products Regulatory Agency” OR “FDA” OR “Food and Drug Administration” OR “Royal Pharmaceutical Society” OR “National Health Service” OR “NHS” OR “Department of Health”
Effects	Cost and cost analyses, time-to-treatment	Consequence* OR change* OR outcome* OR effect* OR implication* OR result* OR opinion* OR cost* OR delay* OR “customs union” OR “free trade” OR “severe” OR “severity”

### Screening and Article Selection

The citation management software Mendeley will be used to store articles and remove duplicates before screening. The titles and abstracts of all identified articles and grey literature sources will be screened by two independent reviewers to minimize selection bias. As grey literature does not always have an abstract, summaries or tables of contents will be screened for eligibility instead [17]. The full texts of articles and grey literature will then be read to determine final inclusion in the review. Disagreements between reviewers at both initial and full-text screening stages will be resolved with discussion. If no consensus can be reached, a third reviewer will make the final decision. Details of the screening and selection process will be recorded in a PRISMA flow diagram to ensure reproducibility.

### Data Extraction

A table for data extraction will be set up with the predetermined outcomes. The primary outcome will be the expected consequences of Brexit for the UK pharmaceutical supply chain. One reviewer will perform the data extraction from all of the papers, and a second reviewer will check the data extraction for all the full texts. Disagreements between the two reviewers will be resolved by discussion, and a third reviewer will be consulted if consensus cannot be reached. An initial review of the literature has suggested items to be extracted (Textbox 1), but other data identified during the review will be included if relevant.

**Data to be extracted from articles.**

**General study information**

- Date of publication
- Type of source (eg, peer-reviewed article, institute report, press release)
- Demographics of authors (anything reported, including location, academic affiliation or workplace, age)

**Effects of Brexit**

- What form(s) of Brexit is (are) being considered?
- Consequences for the United Kingdom identified in the publication, including but not limited to the following: estimated costs of Brexit, estimated delays in production or availability of drugs, impact of delays and availability on health outcomes
- Reasons provided for expected consequences
- Severity of consequences expected by author(s)

### Quality Appraisal and Risk of Bias Assessment

After the final selection of the studies, the risk of bias and quality of sources will be independently assessed by two reviewers. Disagreements in judgment will be discussed before consulting a third reviewer. Any randomized controlled trials that are included in the review will be assessed using the Cochrane Collaboration Risk of Bias tool [18]. The quality of other types of studies will be assessed using the relevant CASP checklists [19]. The quality of grey literature sources will be assessed using the AACODS checklist [15].

### Data Analysis and Synthesis

Eligible sources will subsequently be reviewed in detail and data will be extracted, categorized, and recorded in a predesigned Excel spreadsheet (Microsoft Corp). A meta-analysis or statistical analysis is unlikely to be feasible, due to the anticipated variety of source types and reported outcomes, so we will conduct a descriptive analysis to summarize the extracted data. The discussion will synthesize the data to summarize the currently expected consequences of Brexit on the UK pharmaceutical supply chain and will assess the degree of agreement on the likelihood and severity of these consequences.

## Results

The results will be included in the scoping review, which we aim to publish in 2020. The expected consequences reported by the publications (and their predicted severity) are anticipated to be conditional on the publications’ expectations of the form Brexit will take (ie, how Brexit will unfold and what deal, if any, will be reached).

## Discussion

A systematic and scoping review of academic and grey literature will increase clarity on the different ways Brexit might impact the UK pharmaceutical supply chain and the degree of agreement on the likelihood and potential severity of those consequences. As the situation around Brexit has been highly variable and unstable since the referendum in 2016, more recent publications (as well as those of higher quality) will be weighed more heavily when drawing conclusions. Additionally, any trends identified in how expected consequences have changed over time will be discussed in the context of the key points of the Brexit negotiations.

Understanding the potential consequences and their likelihood could help improve preparation for Brexit and could inform decisions on how to manage the change from the status quo to whatever the new relationship with the EU becomes. In the short-term, this has particular benefit for scientific advisors, negotiators, and policy makers, but in the long-term, the decisions they make when establishing the structure for new UK-EU relations will have significant effects on all stages of the pharmaceutical supply chain, which in turn will affect UK pharmaceutical companies, health care systems, and residents. Based on the data, this section will explore what conclusions can be drawn and with what degree of confidence, the limitations of the scoping review, and what directions future research should take.

## Abbreviations

AACODS: Authority, Accuracy, Coverage, Objectivity, Date, Significance
CASP: Critical Appraisal Skills Programme
EMA: European Medicines Agency
EU: European Union
HMIC: Healthcare Management Information Consortium
MHRA: Medicines and Health Products Regulatory Agency
WTO: World Trade Organization
